# A cognitive-affective dual-path model of learner engagement in AI-supported writing feedback

**DOI:** 10.3389/fpsyg.2026.1868103

**Published:** 2026-07-15

**Authors:** Ting Li, Azlina Abdul Aziz, Nur Ainil Sulaiman

**Affiliations:** Faculty of Education, National University of Malaysia (UKM), Bangi, Malaysia

**Keywords:** artificial intelligence in education, automated writing evaluation, cognitive and affective processes, L2 writing, learner engagement

## Abstract

As artificial intelligence (AI) becomes increasingly integrated into educational contexts, understanding how learners engage with AI-generated feedback is critical. Although prior research has examined the effectiveness of automated writing evaluation, less attention has been given to the cognitive and affective processes through which learners interpret and respond to such feedback. This study employed a constructivist grounded theory approach to investigate how Chinese undergraduate EFL learners engaged with AI-supported feedback during L2 writing revision. Participants were 24 English-major undergraduates at a public university in China. Data were generated through six semi-structured small-group interviews and analyzed through iterative open, axial, and selective coding. The analysis identified three interrelated domains—cognitive engagement, contextual evaluation, and affective regulation—which were integrated into an interpretive cognitive–affective dual-path model. When feedback was perceived as transparent, manageable, meaning-preserving, and aligned with task demands, learners demonstrated stronger cognitive engagement, greater willingness to experiment with complex structures, and sustained motivation. In contrast, feedback perceived as opaque, overly evaluative, or misaligned with learner expectations was associated with negative affect, selective uptake, risk avoidance, and strategic simplification. Learner evaluative filtering appeared to shape how noticing developed into revision action. These findings provide a process-oriented account of learner engagement in AI-supported writing environments and suggest that the influence of AI-generated feedback is filtered through learners' evaluative judgement, contextual appraisal, and affective regulation.

## 1 Introduction

In technology-enhanced learning environments, artificial intelligence (AI) has increasingly reshaped how learners engage with feedback and regulate their learning processes. In Chinese university EFL classrooms, a recurring pattern is that learners can explain grammatical rules yet struggle to produce structurally varied sentences in extended academic writing. This gap between declarative knowledge and actual performance becomes particularly salient in complex writing tasks. AI-supported writing feedback has been introduced as a practical response, offering immediate feedback on grammar, wording, and sentence structure. However, learners' experiences with such feedback are not uniform: while some learners use it to support structural experimentation, others report confusion, anxiety, or strategic simplification, suggesting that learners' responses to AI-supported feedback are shaped by underlying cognitive and affective processes.

In automated writing evaluation (AWE) contexts, learners' engagement with feedback is influenced by how they interpret algorithmic scoring, diagnostic rules, and interface-based feedback during revision. AWE refers to systems that analyze learner writing and generate evaluative scores and/or formative feedback on linguistic and rhetorical features (e.g., grammar, lexis, cohesion, and style). In this article, automated feedback is used as an umbrella term for such system-generated responses, distinguished from automatic feedback, which involves rule-triggered alerts without explanatory scaffolding. This distinction matters because learners' responses depend not only on feedback content but also on how it is perceived and evaluated, which in turn influences engagement and learning behavior.

Automated writing evaluation has been widely examined as a means of supporting noticing, revision, and self-regulated learning. Prior research has shown that AWE can support grammatical accuracy and textual refinement, but learners' responses remain shaped by feedback design, task context, and their interpretation of feedback value and relevance (e.g., Ranalli, 2021; Stevenson and Phakiti, 2014). Importantly, this research consistently shows that learners do not simply apply feedback; rather, they actively filter, trust, resist, or adapt it, indicating that feedback uptake is shaped by learner engagement processes.

Research on second language writing further shows that structural development is non-linear and fluctuates in response to task demands and cognitive load (Larsen-Freeman, 2012; Verspoor et al., 2012; Yang and Lu, 2021; Révész, 2023). While such variation has traditionally been interpreted as a feature of linguistic development, in the present study it is treated as an observable outcome of underlying learner engagement processes.

Despite the potential benefits of automated feedback, it remains unclear whether such systems promote deeper learning or merely reinforce surface-level revision strategies. Evidence suggests that learners selectively engage with feedback, prioritizing suggestions that are easy to interpret or directly linked to evaluation, while avoiding those perceived as complex or uncertain (Zheng and Yu, 2018; Zhang and Hyland, 2022). Affective responses play a critical role in this process. During preliminary analysis, learners frequently reported frustration, anxiety, and loss of confidence when encountering unclear or difficult feedback. According to Control-Value Theory (Pekrun, 2006), such emotions are shaped by learners' perceptions of control and task value. When learners feel capable of understanding and acting on feedback, they are more likely to engage with challenging revisions; when they do not, they tend to withdraw or adopt simplified strategies. Similar patterns have been reported in studies on feedback and writing motivation (Han and Hyland, 2019; Yu et al., 2020; Vyatkina, 2015).

Despite growing interest in automated feedback, several gaps remain. First, much of the existing research relies on product-level comparisons, offering limited insight into learners' cognitive and affective processes. Second, affective responses are often treated descriptively rather than integrated into explanatory models of learner engagement. Third, although learner proficiency and task characteristics are known to influence performance, their interaction with engagement processes in AI-supported contexts remains underexplored.

To address these gaps, this study adopts a grounded theory approach to examine how university learners engage with AI-supported feedback during revision. Rather than asking whether feedback “works,” the study focuses on how learners interpret, evaluate, and respond to feedback, and how these processes shape learning behavior. Specifically, it investigates: (1) how learners engage with AI-supported feedback; (2) what cognitive and affective processes emerge during feedback interpretation; and (3) how learner proficiency and task characteristics influence these processes.

Based on inductive analysis, the study proposes a cognitive-affective dual-path model in which AI-supported feedback can either facilitate or constrain learner engagement. From an educational psychology perspective, AI-supported feedback systems can be understood as engagement environments in which learning behavior is shaped by the interaction of cognitive processing, contextual evaluation, and affective appraisal. By situating automated writing evaluation within a learner engagement framework, this study offers a process-oriented account of learning behavior in technology-enhanced environments.

## 2 Literature review

### 2.1 Structural development in L2 writing

Syntactic complexity has traditionally been regarded as a central dimension of L2 writing development because it reflects learners' ability to construct structurally varied and hierarchically organized sentences (Ortega, 2003; Manchón, 2020). Subsequent research has shown that complexity is not a unitary construct but multidimensional, encompassing features such as length, subordination, coordination, and phrasal elaboration (Lu, 2011; Bulté and Housen, 2012; Kyle and Crossley, 2018). Later studies further demonstrated that different indices capture distinct developmental trajectories, and that short-term instructional or feedback effects may selectively influence specific dimensions rather than overall complexity (Bulté and Housen, 2014). More recent work also highlights the role of task type, task complexity, proficiency level, and genre in shaping how complexity manifests in learner writing (Kuiken and Vedder, 2007; Robinson, 2001; Bulté and Housen, 2020).

In the present study, syntactic complexity is not treated as an isolated linguistic construct but as an observable outcome of learner engagement processes. While traditional perspectives distinguish complexity from grammatical accuracy—viewing accuracy as error reduction and complexity as structural expansion—this distinction is particularly relevant in automated feedback contexts, where feedback tends to prioritize correctness while offering limited support for deeper structural development.

From a dynamic systems perspective, structural development is inherently non-linear and adaptive rather than cumulative. Learners' use of complex structures fluctuates in response to task demands and cognitive load, often involving periods of growth, stagnation, and temporary regression (Larsen-Freeman, 2012; Verspoor et al., 2012; Vyatkina, 2015; Révész, 2023). Such variation has typically been interpreted as a feature of linguistic development. However, it can also be understood as reflecting shifts in learners' cognitive engagement under varying conditions.

Corpus-based research further supports the uneven nature of structural development. Norris and Ortega (2009) showed that different indices develop at different rates, while Lu (2011) found that learners often rely on relatively simple structures even at advanced levels. Studies in the Chinese university context similarly demonstrate that task demands and genre strongly shape learners' structural choices, with some dimensions developing more readily than others (Yang and Lu, 2021; Yang et al., 2015). These findings suggest that structural development is closely tied to contextual and cognitive factors that influence how learners engage with writing tasks.

Taken together, this body of research indicates that structural complexity should not be viewed solely as a linguistic outcome, but as a manifestation of underlying learner engagement processes shaped by cognitive demands and contextual conditions.

### 2.2 Feedback literacy and learner agency in AI-supported writing

Feedback in writing development is increasingly understood as a learner-centered process involving interpretation, evaluative judgement, and action (Carless and Boud, 2018; Boud and Molloy, 2013). In AI-supported writing, learners encounter immediate, system-generated feedback, but its usefulness depends on their capacity to interpret, evaluate, and act upon it. Learners actively judge whether suggestions preserve intended meaning, fit the task or genre, or improve text quality, demonstrating feedback literacy and internal/self-feedback processes (Han and Hyland, 2015; Carless and Boud, 2018).

Recent empirical work highlights the influence of feedback source. Teacher, peer, and AI feedback differ in perceived authority, contextual sensitivity, dialogic quality, and motivational impact. Studies show that students rely on teacher feedback for content and complex rhetorical issues, while using AI feedback primarily for surface-level language or organizational support (Pozdniakov et al., 2026; Sperber et al., 2025; Weidlich et al., 2025). These studies demonstrate that students' evaluative judgement is sensitive to the feedback source and that AI feedback is most effective when it complements rather than replaces human-mediated guidance.

In the present study, feedback literacy and learner agency are used to interpret how Chinese undergraduate EFL learners engaged with AI-supported writing feedback. Learners did not follow AI suggestions mechanically; rather, they filtered, evaluated, and adapted suggestions according to their own goals, knowledge, and task expectations. This framework helps explain why similar AI-generated feedback may support structural experimentation and confidence for some learners, while producing uncertainty, resistance, or strategic simplification for others.

### 2.3 Automated feedback as an interpretive tool for learning

Automated writing evaluation (AWE) tools have been widely adopted in L2 writing instruction due to their capacity to provide immediate and individualized feedback. These systems typically focus on grammatical accuracy, lexical choice, and sentence-level form, and are often used to complement teacher feedback. Prior research has shown that automated feedback can support improvements in grammatical accuracy, particularly among lower-proficiency learners (Bitchener and Ferris, 2012; Ranalli, 2021).

From a learner engagement perspective, however, AWE systems function not merely as sources of corrective input but as mediational tools that shape how learners interpret, prioritize, and respond to feedback. Their design—including scoring mechanisms, feedback presentation, and interface features—influences how feedback is perceived and enacted during revision.

Commonly used tools such as Grammarly rely on rule-based and statistical models to detect surface-level issues, while platforms such as Pigai.org, widely used in Chinese tertiary EFL contexts, provide holistic scores alongside localized feedback. These evaluative features often guide learners' attention and revision priorities, but may also encourage score-oriented engagement rather than deeper learning.

Empirical findings regarding the impact of automated feedback on structural development remain mixed. Some studies report increased awareness of sentence structure, whereas others show that learners engage selectively, prioritizing feedback that is easy to interpret or directly linked to scoring outcomes (Zheng and Yu, 2018). This suggests that automated feedback is not passively applied but actively filtered through learners' engagement processes.

Qualitative studies further highlight the role of learner agency. Learners may resist automated suggestions when these conflict with intended meaning or rhetorical goals (Zhang and Hyland, 2022), and their trust in feedback systems strongly shapes their willingness to revise (Han and Hyland, 2019). When feedback is perceived as reliable and relevant, learners are more likely to engage in substantive revision; when it is perceived as opaque or overly evaluative, engagement tends to remain superficial.

Taken together, these studies indicate that the effects of automated feedback depend not only on its technical capabilities but also on how it is perceived, interpreted, and negotiated by learners. In this sense, AWE systems are best understood as mediational tools whose effects on learning are filtered through learners' engagement processes.

### 2.4 Learner engagement and cognitive-affective processes

To understand how automated feedback shapes learning, it is necessary to conceptualize learner engagement as a multidimensional process involving cognitive, behavioral, and affective components. Research on feedback literacy suggests that learners' uptake of feedback depends not only on feedback quality but also on their capacity and willingness to engage with it (Carless and Boud, 2018).

Several theoretical perspectives inform this process (Schmidt, 1990). Noticing Hypothesis highlights the role of attention to form, while Swain's (2005). Output Hypothesis emphasizes production and hypothesis testing. Although these frameworks account for key cognitive mechanisms, they provide limited explanation for why learners sometimes disengage from feedback despite noticing it or having opportunities for output.

From an educational psychology perspective, affective appraisal plays a critical role in regulating engagement. Control-Value Theory (Pekrun, 2006) suggests that learners' emotional responses are shaped by their perceived control and task value. In automated feedback contexts, these perceptions influence whether learners sustain or withdraw cognitive engagement. Empirical studies support this view, linking negative emotions to avoidance strategies and reduced willingness to engage with challenging feedback (Yu et al., 2020).

Beyond achievement-emotion accounts, learner engagement with automated feedback is also shaped by motivational regulation and technology acceptance. From a self-determination perspective, environments that support autonomy and competence are more likely to sustain engagement, whereas controlling or opaque evaluation may undermine perceived agency (Deci and Ryan, 2002). In addition, learners' willingness to take risks is influenced by value appraisals and future-self orientations (Dörnyei and Ushioda, 2009), while perceived usefulness and ease of use further shape engagement in technology-mediated contexts (Venkatesh et al., 2003).

Despite these insights, several gaps remain. First, many studies rely on product-level comparisons, offering limited insight into learners' underlying cognitive and affective processes. Second, affective responses are often acknowledged descriptively rather than integrated into explanatory models of learner engagement. Third, although proficiency level and task characteristics are known to influence performance, their interaction with engagement processes in AI-supported feedback contexts remains underexplored.

Taken together, these perspectives highlight the need for an integrated framework that explains how cognitive processing and affective regulation jointly shape learner engagement with AI-supported feedback. This gap motivates the present study's process-oriented approach.

## 3 Research design

### 3.1 Constructivist grounded theory approach

This study adopted a constructivist grounded theory approach to examine how Chinese undergraduate EFL learners engaged with AI-supported feedback during feedback-mediated revision. Grounded theory was selected because the research focus was not on measuring the effectiveness of automated feedback, but on understanding how learners interpreted feedback and how these interpretations shaped learning decisions. This inductive and iterative orientation is consistent with the foundational principles of grounded theory and its subsequent methodological development (Glaser and Strauss, 1967; Corbin and Strauss, 2008; Charmaz, 2014). During the early stages of the research, it became evident that existing feedback frameworks did not fully capture the diversity of learners' responses, particularly when affective reactions were involved. An inductive approach was therefore appropriate.

The study followed Charmaz's (2014) constructivist orientation, which views analysis as co-constructed through interaction between researchers and participants. Learners' accounts were treated as situated interpretations shaped by task demands, institutional context, and prior experience with automated feedback systems. Rather than seeking objective causal explanations, the analysis focused on how learners made sense of feedback and how this sense-making informed their engagement behavior. Accordingly, the model developed in this study is presented as an interpretive explanatory account of learners' reported engagement processes, rather than as a causal model of feedback effects.

Researcher reflexivity was maintained throughout the analysis. As the researchers had prior experience in EFL writing instruction, automated feedback, and language education research, reflexive memos were used to monitor how existing assumptions about AI-supported feedback, learner engagement, and writing development might shape interpretation. Coding decisions, category definitions, and emerging theoretical relationships were discussed among the research team. This reflexive process was consistent with the constructivist grounded theory position that findings are not simply discovered from the data, but are interpretively constructed through sustained engagement with participants' accounts, analytic comparison, and researcher reflexivity. Grounded theory was therefore particularly suitable for this study because it enabled close examination of learners' meaning-making processes as they interacted with AI-supported feedback within an educational technology environment.

### 3.2 Participants, data collection, and analysis

As shown in Table 1, participants were 24 second-year English-major undergraduates at a public university in Shanxi Province, China. Purposive sampling was used to ensure that all participants had sustained experience using automated feedback tools in writing coursework. Learners' general English proficiency was indexed using CET-4 scores and instructors' holistic evaluations. For analytic comparison, participants were grouped into high-, mid-, and low-proficiency categories, corresponding broadly to CEFR B2, B1–B2, and B1 levels.

**Table 1:** Summarizes participants' background characteristics.

Variable	Description
Number of participants	24 English-major undergraduates
Gender	17 female, 7 male
Age range	Approximately 19–21 years
Years of English learning	Approximately 12 years (formal instruction)
English proficiency indicator	CET-4 scores ranging from pass (425) to 602
Proficiency grouping	High, Mid, Low (based on CET-4 performance and instructor evaluation)
Automated feedback tools used	Pigai.org (n = 24); Grammarly (n = 14)
Patterns of AWE use	Course-mandated use of Pigai; supplementary use of Grammarly
Typical context of use	University writing assignments and revision tasks

CET-4 scores (total score = 750) were used as a reference indicator of general English proficiency and were supplemented by instructors' holistic evaluations to form proficiency groupings for qualitative analysis.

All participants reported regular use of Pigai.org, and 14 participants also used Grammarly independently. Pigai.org was primarily associated with course requirements and score-oriented revision, whereas Grammarly was more often described as a supplementary tool for sentence-level refinement. Learners' experiences with both systems provided a basis for examining how feedback characteristics influenced engagement. Pigai.org was selected as the focal institutional tool because it was course-mandated and embedded in participants' assessment ecology, making its scores and feedback consequential for learning decisions. Grammarly was included as a complementary system because some participants used it voluntarily for sentence-level refinement, allowing comparison between a score-salient, institutionally positioned tool and a self-initiated, form-focused tool. This combination was theoretically aligned with the study's aim to examine how feedback characteristics (e.g., scoring salience, transparency, and genre fit) interact with learners' cognitive-affective engagement during feedback-mediated revision.

Data were collected through six semi-structured small-group interviews (approximately 30 min each). The small-group format was adopted not to elicit consensus, but to facilitate interactional recall and elaboration, enabling participants to articulate reasoning, comparisons, and affective responses that might remain implicit in one-to-one interviews. All sessions were audio-recorded, transcribed verbatim, and analyzed using constant-comparative coding procedures consistent with constructivist grounded theory. The interview protocol focused on learners' experiences with AI-supported feedback, including how they interpreted suggestions, decided whether to revise, and responded emotionally to feedback. Interaction patterns varied across proficiency groups. Higher-proficiency groups generally displayed more balanced participation, whereas lower-proficiency groups showed greater hesitation. These patterns were treated as analytically meaningful rather than as methodological limitations, as they provided additional insight into how learners' confidence, linguistic resources, and willingness to articulate evaluative judgements shaped their feedback engagement.

Coding followed an iterative process of constant comparison consistent with constructivist grounded theory. Initial coding involved close examination of transcripts to identify meaning units related to learners' feedback interpretation, cognitive processing, contextual evaluation, affective responses, and revision decision-making. *In vivo* codes were retained where participants' wording captured nuanced evaluations, such as uncertainty about scoring logic, concern about meaning distortion, selective acceptance of automated suggestions, or preference for human explanation. Through repeated comparison, similar codes were merged into higher-level first-order categories, including error noticing, structural experimentation, score-oriented revision, scoring opacity, meaning-preservation judgement, genre/context-fit judgement, confidence, frustration, selective uptake, and resistance. Axial coding then examined relationships among these categories and organized them into broader analytic domains: cognitive engagement, contextual evaluation, and affective regulation. Selective coding integrated these domains into a coherent explanatory account, leading to the identification of two contrasting engagement pathways: a facilitative pathway and a constraining pathway.

To strengthen analytical transparency during revision, a supplementary coding audit was conducted. This audit did not replace the original grounded theory analysis; rather, it re-examined the six focus-group transcripts to clarify how the transition from initial codes to conceptual categories and model construction was made. Particular attention was given to learners' feedback literacy-oriented evaluative judgements and internal/self-feedback processes. Segments in which learners interpreted, questioned, accepted, resisted, or adapted automated feedback were marked using abbreviated codes across five analytic families: cognitive engagement (e.g., C-EN for error noticing; C-SE for structural experimentation), feedback literacy-oriented judgement (e.g., FL-CJ for comprehensibility judgement; FL-MJ for meaning-preservation judgement), internal feedback (e.g., IF-IM for internal comparison with intended meaning; IF-TG for internal comparison with task or genre expectations), contextual evaluation (e.g., CT-SO for scoring opacity; CT-LE for lack of explanation), and affective regulation (e.g., AF-CF for confidence; AF-RS for resistance). Pathway relevance was also marked as facilitative (FP), constraining (CP), or mixed (MX).

The coding abbreviation system was used as an audit-trail tool rather than as a set of predetermined variables. For example, statements about noticing grammar or sentence-structure problems were coded under cognitive engagement; statements questioning whether automated suggestions were accurate, genre-appropriate, or meaning-preserving were coded under feedback literacy-oriented judgement; and statements comparing AI feedback with intended meaning, prior knowledge, task expectations, scoring consequences, or ability to revise were coded as internal feedback generation. These coded segments were then compared across groups and linked to facilitative, constraining, or mixed engagement pathways. The full coding abbreviation system and transcript-level examples were retained in the supplementary audit trail.

Several strategies were used to enhance analytic rigor and trustworthiness (Lincoln and Guba, 1985). Two trained researchers coded the data independently, and discrepancies were resolved through discussion and by returning to the transcript evidence. Analytic memos documented coding decisions and emerging interpretations. The fifth focus group reached theoretical saturation, as no substantially new categories emerged. Grounded theory was particularly suitable for this study because it enabled close examination of learners' meaning-making processes as they interacted with AI-supported feedback within an educational technology environment. The supplementary audit further supported analytical transparency by retaining transcript-level coding traces and linking representative excerpts to the final categories and pathways.

Researcher reflexivity was maintained throughout the analysis. As the researchers had prior experience in EFL writing instruction, automated feedback, and language education research, reflexive memos were used to monitor how existing assumptions about AI-supported feedback, learner engagement, and writing development might shape interpretation. This reflexive process was consistent with the constructivist grounded theory position that findings are interpretively constructed through sustained engagement with participants' accounts, analytic comparison, and researcher reflexivity.

## 4 Category extraction and model construction

### 4.1 Open coding: emergent patterns in learners' feedback engagement

Open coding aimed to capture how learners described their experiences with automated feedback during syntactic revision. Line-by-line coding was conducted to remain close to participants' wording and to identify evaluative and affective cues embedded in their accounts. At this stage, the focus was on mapping the range of learner responses rather than imposing predefined categories.

Early coding revealed that learners rarely discussed feedback in purely technical terms. References to sentence structure were frequently accompanied by judgments about usefulness, trust, and emotional reactions. *In vivo* codes were therefore retained where participants' expressions reflected these layered experiences, such as comments on unclear scores, fear of meaning distortion, uncertainty about revision value, or deliberate simplification. These expressions provided early evidence that learners were not passive recipients of automated feedback, but active interpreters of feedback meaning and relevance.

The initial coding process yielded over 600 meaning units. Through constant comparison, infrequent or conceptually weak codes were eliminated, while overlapping codes were merged. This process resulted in 18 first-order categories capturing core aspects of learner experience, including syntactic noticing, revision behavior, evaluation of feedback relevance, and motivational shifts. Representative examples of the first-order categories and their corresponding raw excerpts are presented in Table 2. At this stage, the analysis remained descriptive, establishing a foundation for higher-level integration.

**Table 2:** Examples of first-order concepts generated from open coding.

Open category (first-order concepts)	Representative raw excerpts (concepts)
Feedback-supported error awareness	G2S1: Immediate reminders for spelling, grammar, and punctuation errors. *(basic error awareness)*
	G3S2: Identification of improper wording, inaccurate sentence patterns, and collocational problems. *(enhanced error awareness)*
Form-focused revision guidance	G1S1: Provides phrase-level corrections and sentence-structure optimization suggestions. *(local revision guidance)*
	G5S4: Recommends cohesive devices to enhance inter-sentential logic and complexity. *(cohesion-oriented revision prompt)*
Immediate feedback engagement	G1S3: Errors appear instantly, enabling targeted revision. *(immediate revision response)*
	G5S2: Learners adjust sentences immediately after receiving prompts. *(rapid feedback uptake)*
Lexico-syntactic refinement engagement	G4S1: Highlights collocation issues and grammatical misuse. *(lexical–grammatical refinement)*
	G5S4: Recommends more appropriate or advanced expressions. *(advanced expression refinement)*
Structural integration support	G5S3: Suggests combining two simple sentences into a more rigorous complex structure. *(sentence integration support)*
	G6S1: Recommends adding cohesive devices to enhance logical flow. *(coherence-oriented structural support)*
Scaffolded engagement with complexity	G1S2: Provides explicit suggestions for making sentences more complex and using more advanced wording. *(guided complexity attempt)*
	G2S4: Encourages use of subordinate clauses and cohesive devices. *(confidence to attempt complexity)*
Perceived opacity and scoring uncertainty	G4S3: Lack of transparency in scoring criteria creates a “black box” effect. *(perceived scoring opacity)*
	G2S2: Unclear scoring logic for complex sentences leads to wording confusion. *(unclear scoring criteria)*
Score-driven revision bias	G3S3: High scores depend mainly on grammatical accuracy rather than syntactic complexity. *(grammar-weighted scoring)*
	G6S2: Scores plateau despite complex sentence attempts. *(restricted score improvement)*
Simplified evaluation bias	G2S2: Scoring prioritizes grammar and vocabulary over content quality. *(surface-feature evaluation)*
	G3S4: Rankings fail to reflect actual writing proficiency. *(perceived evaluation mismatch)*
Motivational decline after feedback plateau	G1S3: No further suggestions after several revisions. *(feedback plateau)*
	G6S2: Score stagnation reduces motivation for continued revision. *(reduced revision persistence)*
Academic register engagement	G3S4: The tool effectively boosts complex sentence use in academic tasks. *(academic-register support)*
	G5S2: Enhances accuracy and overcomes lexical limitations in academic writing. *(domain-specific language support)*
Strategic engagement transfer (high-proficiency)	G5S2: Learners imitate and transfer high-quality structures from feedback. *(adaptive structure transfer)*
	G6S3: Feedback suggesting alternative forms can be remembered and used in later writing. *(future writing transfer)*
Selective engagement (mid-proficiency)	G2S3: Learners only revise suggestions they can understand. *(uptake of understandable feedback)*
	G3S1: Unfamiliar phrases are rarely applied in later writing. *(limited uptake of unfamiliar feedback)*
Risk-avoidant engagement (low-proficiency)	G3S2: Suggestions are difficult, leading to sentences that do not match intended meaning. *(meaning distortion concern)*
	G1S3: Modifications distort meaning; reasons for suggestions unclear. *(risk-avoidant structure use)*
Genre-related feedback constraints	G1S4: Not suitable for genres such as reading responses or prose. *(genre mismatch)*
	G2S3: Learners must judge whether suggestions fit the target genre. *(genre-fit judgement)*
Limited cross-genre and whole-text support	G5S4: Effective for academic writing but less helpful for other genres. *(restricted cross-genre support) G3S1: Automated feedback is basic and cannot properly evaluate subjective ideas. (limited whole-text evaluation)*
Feedback-driven engagement activation	G2S2: Instant feedback encourages more active writing. *(feedback visibility as motivation)*
	G5S4: Anticipation of score improvement forms a positive cycle. *(score-related motivation)*
Score-oriented strategic revision	G4S2: Number of revisions driven by scores. *(performance-driven revision)*
	G4S3: Learners “pile up” complex sentences to boost scores. *(strategic complexity manipulation)*

GS denotes participant ID (Group ^*^-Student ^*^). Concepts in parentheses represent initial open-code labels derived from representative raw statements.

A key analytic decision during open coding was to treat learners' strategic behavior as analytically meaningful. Statements about revising primarily for scores, accepting only understandable suggestions, resisting feedback that distorted intended meaning, or avoiding complex structures initially appeared to reflect individual preferences. However, their recurrence across proficiency groups indicated that such strategies represented systematic responses to perceived feedback conditions, including scoring opacity, limited explanation, genre mismatch, and perceived risk. These patterns later informed the interpretation of feedback literacy-oriented judgement and internal comparison during axial and selective coding.

### 4.2 Axial coding: relationships among cognitive, contextual, and affective dimensions

The next analytic phase focused on integrating first-order categories by examining patterns of co-occurrence and functional similarity. In line with the revised coding structure, axial coding also examined conceptual relationships among cognitive engagement, contextual evaluation, affective regulation, and learners' evaluative judgement of feedback. Categories related to noticing structural issues, interpreting feedback, and attempting revision were grouped as cognitive engagement. These categories captured how learners used automated feedback to identify linguistic problems, refine local expression, and experiment with more complex or coherent structures. In contrast, categories describing unclear scoring logic, grammar-weighted evaluation, and genre mismatch were grouped as contextual constraints, as they shaped the conditions under which learners engaged with feedback.

Affective responses intersected with both domains. Learners' expressions of confidence, frustration, anxiety, or hesitation were closely linked to how they evaluated feedback and decided whether to revise. Rather than treating affect as a by-product of feedback, the analysis interpreted affective regulation as part of the process through which learners sustained, redirected, or limited their engagement. This wording positions affective regulation as an interpretive pattern in learners' accounts rather than as a deterministic mediating mechanism. This shift marked an important analytic turning point, as affect moved from a secondary theme to a central explanatory dimension.

A further pattern emerged around learners' evaluative judgement of feedback. Categories such as perceived opacity and scoring uncertainty, selective engagement, risk-avoidant engagement, genre-related feedback constraints, and score-oriented strategic revision indicated that learners actively judged whether automated feedback was understandable, accurate, useful, meaning-preserving, score-relevant, and appropriate to the task or genre. These judgements cut across the cognitive, contextual, and affective domains. For example, a learner might notice a sentence-level suggestion cognitively, question its accuracy or genre fit contextually, and then respond with confidence, frustration, resistance, or selective uptake.

Through this integrative process, the initial 18 categories were reorganized into a smaller set of core categories aligned with three interrelated domains: cognitive engagement, contextual evaluation, affective regulation. The relationships among the three core domains, eight second-order themes, and first-order categories are summarized in Table 3. In addition, learners' evaluative judgement and internal comparison were observed across these domains and helped explain how they interpreted automated suggestions before accepting, adapting, resisting, or avoiding them. These domains captured not only learners' actions, but also the conditions and emotional responses shaping their engagement behavior. This analytic refinement provided the basis for the selective coding stage, where the three core domains were integrated with learners' evaluative filtering into an interpretive cognitive-affective dual-path model.

**Table 3:** Axial coding results: core domains, second-order themes, and first-order concepts.

Dimension (core domain)	Axial category (second-order themes)	Open category (first-order concepts)	Category description
Cognitive engagement	Accuracy-oriented feedback engagement	Feedback-supported Error Awareness; Form-focused Revision Guidance; Immediate Feedback Engagement; Lexico-syntactic Refinement Engagement	Represents learners' engagement with automated feedback as a source of linguistic awareness and local revision support. These categories show how learners noticed errors, responded to prompts, and refined grammar, vocabulary, collocation, and sentence-level expression.
	Complexity-oriented engagement awareness	Structural Integration Support; Scaffolded Engagement with Complexity; Academic Register Engagement; Strategic Engagement Transfer	Represents learners' engagement with syntactic complexity and structural development. These categories show how feedback was perceived as supporting sentence integration, academic register development, coherence, and transfer of feedback-supported structures to later writing.
Contextual evaluation	Assessment-driven feedback constraints	Perceived Opacity and Scoring Uncertainty; Score-driven Revision Bias; Simplified Evaluation Bias; Motivational Decline after Feedback Plateau	Represents learners' evaluation of the assessment conditions surrounding automated feedback. These categories show how unclear scoring, grammar-weighted evaluation, surface-oriented metrics, and score plateau shaped learners' judgement of feedback value and revision decisions.
	Genre and whole-text constraints	Genre-related Feedback Constraints; Limited Cross-genre and Whole-text Support	Represents learners' evaluation of feedback relevance across writing contexts. These categories show how learners judged whether automated suggestions fit genre expectations, task purpose, whole-text meaning, and non-academic or literary writing contexts.
	Feedback comprehensibility and relevance judgement	Selective Engagement; Risk-avoidant Engagement; Genre-related Feedback Constraints	Represents learners' judgement of whether feedback was understandable, actionable, meaning-preserving, and contextually appropriate. These categories show how learners accepted, adapted, or resisted feedback according to perceived clarity, relevance, and risk.
Affective regulation	Motivational activation and perceived control	Feedback-driven Engagement Activation; Scaffolded Engagement with Complexity; Academic Register Engagement	Represents positive affective responses that sustained learner engagement. These categories show how feedback visibility, perceived manageability, score anticipation, and perceived control supported confidence, motivation, and willingness to attempt revision.
	Frustration, resistance, and risk management	Motivational Decline after Feedback Plateau; Selective Engagement; Risk-avoidant Engagement; Score-oriented Strategic Revision	Represents negative or protective affective responses that constrained engagement. These categories show how unclear, opaque, difficult, or risky feedback was associated with frustration, resistance, selective uptake, avoidance, or strategic simplification.
	Score-oriented strategic regulation	Score-driven Revision Bias; Score-oriented Strategic Revision; Motivational Decline after Feedback Plateau	Represents learners' affective and strategic responses to score salience. These categories show how learners regulated effort, persistence, and revision strategy according to perceived scoring consequences.

Categories were grouped according to their dominant analytic function during axial coding. Some first-order categories appear across more than one second-order theme because learners' feedback responses often involved overlapping cognitive, contextual, affective, and evaluative dimensions.

Accordingly, axial coding did not treat cognitive, contextual, and affective dimensions as isolated components. Instead, it showed that learner engagement emerged through the interaction of feedback interpretation, contextual evaluation, affective regulation, and internal comparison. This analytic refinement provided the basis for the selective coding stage, in which these relationships were integrated into an interpretive cognitive-affective dual-path model.

### 4.3 Selective coding: integrating engagement pathways into an interpretive dual-path model

The final stage of analysis involved integrating the three core domains—cognitive engagement, contextual evaluation, and affective regulation—into an interpretive dual-path model. Learners' engagement with AI-supported feedback emerged as the central category linking these domains. When learners' accounts were examined holistically, two contrasting but dynamic engagement pathways became apparent. The revised model further clarified that learner evaluative filtering, including feedback literacy-oriented judgement and internal comparison, helped explain how learners moved toward either facilitative or constraining engagement.

In the facilitative pathway, automated feedback was perceived as supporting learner engagement when it enhanced structural awareness, offered understandable revision prompts, and contributed to positive affective responses. Learners reported greater willingness to engage with feedback when it was judged as comprehensible, task-appropriate, meaning-preserving, and manageable. These perceptions supported exploratory engagement, in which learners actively experimented with more complex structures, refined their language use, and transferred feedback-supported forms to later writing. Positive affective responses, such as confidence, motivation, and perceived control, appeared to sustain this pathway, even when revisions involved temporary uncertainty or instability.

In the constraining pathway, similar forms of feedback were associated with reduced or more selective engagement. This pattern emerged when feedback was perceived as opaque, overly score-oriented, difficult to act upon, or misaligned with task or genre expectations. When learners judged feedback as unclear, inaccurate, or likely to distort intended meaning, they often responded with uncertainty, frustration, resistance, or risk awareness. To manage perceived risk, they often shifted toward surface-focused engagement by restricting their responses to easily interpretable corrections, avoiding more complex structures, or simplifying expression. This pattern reflects selective uptake and strategic simplification rather than a simple lack of ability.

Learner proficiency, task characteristics, feedback source, and assessment ecology functioned as moderating conditions across both pathways. Higher-proficiency learners were generally better able to reinterpret feedback, compare automated suggestions with prior linguistic knowledge, and transfer feedback-supported structures across tasks. In contrast, lower-proficiency learners with fewer linguistic resources were more susceptible to cognitive overload, uncertainty, and risk-avoidant revision. Task demands also influenced whether feedback was experienced as supportive or intrusive, particularly when automated suggestions did not align with genre expectations or whole-text meaning. In addition, because Pigai.org was embedded in the assessment ecology, score salience shaped how learners interpreted feedback value and revision priority.

**Figure 1:**
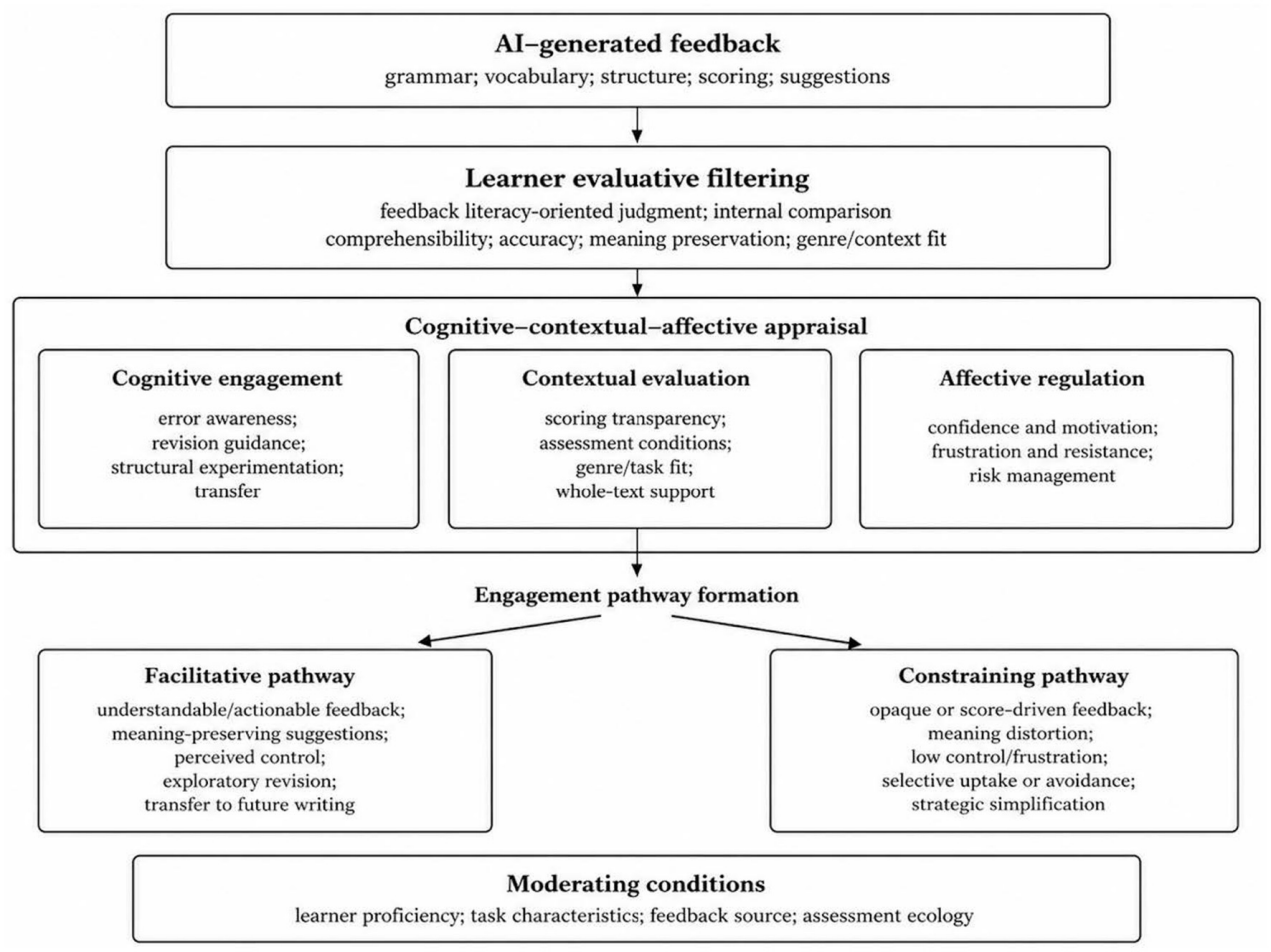
An interpretive cognitive–affective dual-path model of learner engagement with AI-supported feedback. The model presents an interpretive rather than causal account of learner engagement. Learner evaluative filtering refers to feedback literacy-oriented judgement and internal comparison, which operate across the cognitive, contextual, and affective domains rather than as a separate outcome domain. Moderating conditions may shape movement toward either pathway.

These findings were synthesized into an interpretive cognitive-affective dual-path model illustrating how AI-supported feedback may facilitate or constrain learner engagement depending on learners' cognitive engagement, contextual evaluation, and affective regulation. Rather than directly determining learning outcomes, AI-generated feedback was filtered through learners' evaluative judgement and internal comparison before shaping engagement pathway formation. The model therefore highlights that similar feedback inputs may be associated with divergent engagement patterns across learners and contexts. The resulting interpretive cognitive-affective dual-path model is presented in Figure 1.

## 5 Model interpretation and findings

The findings of this study contribute to the growing body of research on learner engagement in AI-supported educational environments by showing how learners interpreted, evaluated, and regulated their responses to automated writing feedback. Rather than treating automated feedback as a purely corrective or linguistic tool, the proposed dual-path model conceptualizes AI-supported feedback as an interpretive engagement environment in which learners' responses are shaped by the interaction of cognitive engagement, contextual evaluation, and affective regulation. This framing is consistent with educational psychology perspectives showing that learning in technology-enhanced environments is shaped not only by task design and feedback provision, but also by learners' perceptions, emotions, and motivational responses.

Drawing on the integrated results of open, axial, and selective coding, this study consolidates 18 first-order categories, eight second-order themes, and three core domains into an interpretive cognitive-affective dual-path model of learner engagement with AI-supported feedback. Rather than viewing automated feedback as a unidirectional source of corrective input, the model shows that learners actively interpret, evaluate, filter, and regulate their responses to feedback. In this sense, AI-supported feedback does not directly determine learning outcomes. Instead, learners' engagement pathways are shaped by how they judge feedback clarity, relevance, meaning preservation, score value, and contextual fit. This interpretation echoes Carless and Boud's (2018) argument that feedback should be understood as a process of learner meaning-making rather than a simple transfer of information, and aligns with research emphasizing learners' active negotiation of feedback in authentic writing contexts (Han and Hyland, 2019).

When cognitive engagement, contextual fit, and affective regulation operate in synergy, AI-supported feedback supports a facilitative pathway characterized by stronger structural awareness, exploratory engagement, and sustained motivation. Conversely, when misalignment occurs—particularly in relation to evaluation transparency, genre or task fit, or proficiency-sensitive processing demands—a constraining pathway emerges, marked by uncertainty, negative affect, and selective or surface-level engagement. To interpret these dual pathways, the present discussion draws on Schmidt's (1990) Noticing Hypothesis, Swain's (2005) Output Hypothesis, and Pekrun's (2006) Control-Value Theory, each of which helps illuminate how cognitive processing, learner evaluative filtering, and affective appraisal jointly shape learner engagement with AI-supported feedback.

### 5.1 Facilitative pathway: from cognitive engagement to exploratory revision

The facilitative pathway identified in this study begins with heightened awareness of structural issues, a process broadly explained by Schmidt's (1990) Noticing Hypothesis. AI-supported feedback increased the salience of structural problems and alternative formulations, enabling learners to recognize gaps between their current writing and more target-like or academically appropriate forms. In this respect, noticing functioned as an important entry point for deeper engagement with feedback.

However, the findings also show that noticing alone was insufficient to ensure meaningful uptake. Learners frequently reported recognizing structural problems but only moving toward substantive revision when feedback was perceived as understandable, transparent, and manageable. This suggests that cognitive engagement was filtered through learners' evaluative judgement before developing into exploratory revision. In other words, awareness became developmentally productive when learners felt capable of acting on the feedback and confident that doing so would support their intended meaning. This interpretation extends Schmidt's framework by showing that, in AI-supported environments, the transition from noticing to uptake is shaped not only by cognitive salience but also by learners' perceived control, evaluative confidence, and judgement of feedback relevance.

The facilitative pathway also resonates with Swain's (2005). Output Hypothesis, which emphasizes hypothesis testing and pushed output in language development. In the present study, learners demonstrated exploratory engagement by attempting restructuring, clause embedding, or more advanced expressions when feedback was interpreted as supportive rather than threatening. Yet the findings also reveal an important boundary condition: opportunities for output were productive only when learners perceived feedback as intelligible, controllable, and aligned with their communicative intentions. When those conditions were present, feedback supported not only revision but also a stronger willingness to experiment with more demanding forms of expression and to transfer feedback-supported structures to later writing.

Taken together, the facilitative pathway is best understood as a cognitive-motivational chain in which structural awareness, evaluative filtering, and exploratory engagement reinforce one another. Automated feedback supported learner engagement not simply by directing attention to form, but by helping learners sustain motivation to engage in cognitively demanding revision under conditions of perceived control, relevance, and meaning preservation. In this way, the model helps explain why AI-supported feedback can promote deeper structural engagement and more confident academic expression for some learners.

### 5.2 Constraining pathway: from contextual misalignment to selective engagement

The constraining pathway emerges when learners encounter contextual and evaluative conditions that limit meaningful engagement with feedback. A major source of difficulty was feedback transparency appraisal. When scoring logic was unclear, or when structurally more ambitious revisions did not lead to expected score gains, learners' trust in the system weakened. This pattern is consistent with Bitchener and Ferris (2012) argument that perceived validity is a precondition for feedback uptake, and with digital feedback research highlighting the negative effects of algorithmic opacity on learner trust (Zheng and Yu, 2018).

Contextual misalignment further intensified these constraints. Automated feedback often appeared to privilege an academic register or specific writing norms, leaving learners to determine whether suggestions were appropriate for the genre or task at hand. Similar genre-tool mismatches have been reported by Zhang and Hyland (2022), who note that feedback optimized for expository prose may not transfer well to narrative or reflective writing. In the present study, this mismatch required learners to filter automated suggestions through their own judgement of task purpose, intended meaning, and genre fit. For lower-proficiency learners in particular, the need to interpret, evaluate and selectively adapt such feedback created substantial cognitive load, reducing the attentional resources available for deeper revision. This is in line with Kormos's (2014) argument that limited attentional capacity constrains linguistic complexity during task performance.

Under these conditions, learners often shifted toward selective engagement or surface-level engagement. Rather than experimenting with more demanding revisions, they focused on easily interpretable corrections or simplified their responses to minimize risk. These patterns were accompanied by negative affective responses such as frustration, anxiety, and resignation. According to Pekrun's (2006) Control-Value Theory, decreased perceived control is closely tied to disengagement, and recent writing research similarly suggests that negative affect narrows attentional scope and encourages risk-avoidant revision behavior (Lee, 2020). In this study, learners frequently defaulted to superficial corrections or reverted to simpler structures in order to preserve meaning stability. This pattern is also consistent with dynamic-systems perspectives, which allow for temporary retreat or simplification under pressure (Verspoor et al., 2012).

Thus, the constraining pathway is best understood as a contextual-affective pattern in which opacity, weak evaluative confidence, and perceived risk were associated with selective engagement. Rather than indicating lack of ability alone, this pathway reflects how learners strategically regulated their effort when feedback was experienced as unclear, misaligned, or emotionally costly. In this sense, selective uptake and simplification were not merely signs of disengagement, but protective responses to feedback that learners judged as difficult to interpret, difficult to trust, or difficult to integrate with their intended meaning.

### 5.3 Integrating the dual pathways

Taken together, the facilitative and constraining pathways clarify how learner engagement with AI-supported feedback is shaped by the dynamic interaction of cognitive, contextual, and affective processes. Rather than treating noticing, output, and emotion as separate or sequential mechanisms, the proposed model shows that these processes are interdependent and differentially activated depending on learners' appraisal of feedback conditions. Learner evaluative filtering helps explain why similar AI-generated feedback may lead to exploratory revision for some learners but selective uptake or simplification for others.

From the perspective of Schmidt's (1990) Noticing Hypothesis, the study confirms that AI-supported feedback can enhance awareness of structural form. However, the dual-path evidence suggests that noticing alone is insufficient to ensure meaningful uptake. High levels of awareness were present in both pathways; what differentiated facilitative from constraining engagement was whether awareness translated into sustained action. This finding refines Schmidt's account by demonstrating that, in AI-supported environments, the transition from noticing to revision depends heavily on learners' perceived control, feedback relevance, and confidence in the feedback system.

Swain's (2005) Output Hypothesis helps explain learners' exploratory engagement in the facilitative pathway, where feedback supported revision as a form of hypothesis testing. At the same time, the present findings identify a clear boundary condition: pushed output does not necessarily support sustained development when feedback is perceived as opaque, misaligned with task demands, or threatening to meaning control. Under such conditions, learners often reduce or abandon revision attempts, suggesting that opportunities for output require sufficient agency, contextual fit and perceived manageability to become developmentally productive.

Pekrun's (2006) Control-Value Theory provides the critical link between these cognitive accounts and learners' affective responses. The present model suggests that affective regulation shapes whether cognitive engagement is sustained or redirected. Positive affect associated with perceived control and task value appeared to support exploratory revision, whereas negative affect was associated with selective, surface-level, or risk-avoidant responses. In this sense, affect is not a secondary by-product of feedback, but part of the appraisal process through which learners decide whether feedback is worth acting upon.

The findings also suggest that AI-supported feedback should be understood within a broader feedback ecology rather than as an isolated technological intervention. Learners' evaluative judgement was shaped not only by feedback content, but also by perceived feedback source, authority, contextual sensitivity, and dialogic potential. Recent research comparing teacher, peer, and AI feedback indicates that learners may attribute different forms of value to different feedback sources: teacher feedback is often associated with contextual interpretation and authority, peer feedback with interaction and reflection, and AI feedback with immediacy and accessibility (Pozdniakov et al., 2026; Sperber et al., 2025; Weidlich et al., 2025). This perspective helps explain why learners in the present study valued AI feedback for rapid sentence-level prompts but also questioned its usefulness when feedback appeared opaque, decontextualized, or insensitive to genre and intended meaning.

Accordingly, the dual-path model does not imply that AI feedback is inherently facilitative or constraining. Rather, it shows that learners' engagement depends on how AI-generated suggestions are interpreted through feedback literacy, internal comparison, contextual appraisal, and affective regulation. AI feedback may support learning when it strengthens noticing, provides manageable revision options, and preserves learners' sense of control. It may constrain engagement when learners perceive it as difficult to interpret, poorly aligned with task or genre expectations, or insufficiently responsive to meaning. This interpretation positions AI-supported feedback as most pedagogically useful when it complements rather than replaces human judgement, peer interaction, and learner reflection.

Overall, the dual-path model extends current theory by showing that AI-supported feedback influences learner engagement not directly, but through interpretive and affective processes that are cognitively initiated, contextually shaped, and affectively regulated. By specifying how these processes interact under different feedback conditions, the model offers a more context-sensitive account of how automated feedback can either support or constrain structural development in L2 writing.

## 6 Conclusion

This study examined how learners engaged with AI-supported feedback during revision. Rather than assuming that automated writing evaluation functions as a uniformly supportive instructional tool, the findings present a more differentiated and process-oriented account of learner engagement. Learners' responses to automated feedback were shaped not only by linguistic competence, but also by how they interpreted feedback cues, evaluated contextual fit, judged meaning preservation, and regulated the affective demands of revision.

By focusing on learners' accounts of feedback use, the study identified two contrasting engagement pathways. In the facilitative pathway, AI-supported feedback promoted learner engagement by enhancing structural awareness, encouraging exploratory revision, and sustaining motivation under conditions of perceived clarity, task relevance, meaning preservation, and manageability. In the constraining pathway, feedback was associated with reduced or more selective engagement when it was experienced as opaque, overly score-oriented, difficult to act upon, or poorly aligned with task demands and genre expectations. Under such conditions, learners often responded with uncertainty, frustration, or selective uptake and surface-level engagement rather than deeper structural experimentation.

A key finding of this study is that awareness alone does not guarantee meaningful uptake in AI-supported feedback environments. Although learners frequently noticed structural problems, this awareness developed into substantive revision only when learners judged feedback as understandable, trustworthy, meaning-preserving, and manageable. When perceived control was weakened because of unclear scoring logic, mismatched feedback difficulty, genre uncertainty, or fear of meaning distortion, learners were more likely to withdraw effort, avoid risk, or simplify their responses. This finding highlights the importance of learner evaluative filtering, through which feedback literacy-oriented judgement and internal comparison shape how learners move from noticing to revision action.

The study also contributes to feedback research by foregrounding learner agency. Learners were not passive recipients of system-generated suggestions; rather, they actively interpreted, filtered, negotiated, and strategically responded to feedback. From this perspective, learning outcomes in AI-supported writing should not be understood as a direct product of feedback exposure. Instead, learners' engagement pathways are shaped by the interaction of cognitive engagement, contextual evaluation, affective regulation, and perceived feedback source. This interpretation suggests that AI feedback may be most pedagogically useful when it complements, rather than replaces, teacher judgement, peer interaction, and learner reflection.

Theoretically, the proposed dual-path model extends current accounts of feedback and learning by integrating cognitive engagement, contextual evaluation, and affective regulation within a single explanatory framework. While the Noticing Hypothesis and the Output Hypothesis help explain how learners attend to and work with feedback, the present findings suggest that their explanatory power in AI-supported environments depends crucially on learners' evaluative judgement, affective appraisal and perceived control. By theorizing feedback uptake as a differentiated engagement process, this study contributes to educational psychology and technology-enhanced learning research by clarifying how AI-supported feedback may either facilitate or constrain learner engagement.

Several pedagogical and design implications follow. Learners, particularly those with fewer linguistic resources, may benefit from explicit guidance in interpreting and evaluating automated feedback, and selectively using automated feedback. Such guidance can support feedback literacy by helping learners judge feedback accuracy, relevance, meaning preservation, and genre fit. From a system-design perspective, greater transparency in scoring logic, clearer explanations, sensitivity to proficiency differences, and stronger genre awareness may reduce unnecessary cognitive and emotional burden while encouraging more sustained and meaningful engagement. In instructional practice, AI-generated feedback should be embedded within a broader feedback ecology that includes teacher support, peer discussion, and opportunities for reflection.

This study has several limitations. The participant group was drawn from a single institutional context, and the findings may not generalize to learners in other disciplines or educational settings. In addition, the study relied on learners' retrospective interview accounts rather than real-time behavioral evidence or systematic analysis of written texts. Future research could address these limitations by examining more diverse learner populations and by combining qualitative interviews with document-based interviews, stimulated recall, revision logs, screen recordings, and corpus analysis of initial and revised drafts. Such designs would allow researchers to connect learners' perceptions of feedback with observable revision behavior and textual development. As AI-supported writing tools continue to evolve, understanding the cognitive and affective dynamics of learner engagement will remain essential for explaining how such systems shape learning in technology-enhanced environments.

## Data Availability

The original contributions presented in the study are included in the article and its Supplementary Material. Further inquiries can be directed to the corresponding author.
